# COVID-19 and Guillain–Barré Syndrome: A Case Report and Review of Literature

**DOI:** 10.3389/fneur.2020.00909

**Published:** 2020-08-21

**Authors:** Antonio Zito, Enrico Alfonsi, Diego Franciotta, Massimiliano Todisco, Matteo Gastaldi, Matteo Cotta Ramusino, Mauro Ceroni, Alfredo Costa

**Affiliations:** ^1^Department of Brain and Behavioral Sciences, University of Pavia, Pavia, Italy; ^2^Clinical Neurophysiology Unit, IRCCS Mondino Foundation, Pavia, Italy; ^3^Laboratory of Neuroimmunology, IRCCS Mondino Foundation, Pavia, Italy; ^4^Unit of Behavioral Neurology, IRCCS Mondino Foundation, Pavia, Italy

**Keywords:** guillain–barré syndrome, miller fisher syndrome, COVID-19, SARS-CoV-2, AMSAN, post-infectious

## Abstract

During the recent coronavirus disease 2019 (COVID-19) outbreak in Northern Italy, we observed a 57-year-old man developing acute motor-sensory axonal neuropathy, a variant of Guillain–Barré syndrome (GBS), 12 days after severe acute respiratory syndrome-coronavirus-2 (SARS-CoV-2) infection. Similarly to other bacterial and viral infections, dysregulation of the immune system due to post-infectious mechanisms, such as the molecular mimicry, could lead to an indirect damage of the peripheral nervous system related to SARS-CoV-2. GBS causes motor dysfunctions that are not easily recognizable in non-neurological settings or in patients requiring ventilatory assistance. Several reports also suggested that GBS and Miller Fisher syndrome (MFS) could be neurological complications of COVID-19. Therefore, we performed a review of the 29 articles so far published, describing 33 GBS cases and five MFS cases associated with SARS-CoV-2 infection. We recommend awareness of this rare, but treatable, neurological syndrome, which may also determine a sudden and otherwise unexplained respiratory deterioration in COVID-19 patients.

## Introduction

The coronavirus disease 2019 (COVID-19) outbreak started at the end of 2019 in Wuhan, the capital of Hubei province, in China. The novel coronavirus was designated as severe acute respiratory syndrome coronavirus 2 (SARS-CoV-2). To date, millions of cases have been confirmed worldwide, and Italy has been one of the most affected countries.

Neurological manifestations have been described in one third of patients with COVID-19. Some of these neurological symptoms have proved to be quite specific, e.g., loss of smell or taste, but other ones are non-specific, e.g., headache, dizziness, or reduced level of consciousness (1). However, whether the neurological symptoms associated with SARS-CoV-2 are attributable to secondary mechanisms (i.e., multiorgan dysfunction or systemic inflammation), an abnormal immune response or the direct injury of the virus is still unknown.

Recently, several case reports have suggested a relationship between the occurrence of Guillain–Barré syndrome (GBS) and a previous SARS-CoV-2 infection, which preceded the GBS onset by up to 4 weeks. Therefore, a post-infectious dysregulation of the immune system, triggered by SARS-CoV2, appears to be the most probable cause.

Conversely, a recent pathological study on postmortem human brain tissues found that anosmia and dysgeusia, described in up to 20% of patients, are more likely due to the direct viral invasion of the olfactory nerve and bulb (2). In addition and in line with this observation, a COVID-19 patient with anosmia showed magnetic resonance imaging (MRI) abnormalities in the olfactory bulb and in the inferior frontal lobe, as a conceivable result of the direct invasion of SARS-CoV-2 through the olfactory pathway *via* trans-synaptic retrograde spreading (3).

Here we describe a patient with an axonal variant of GBS following COVID-19, and we review the available reports in the literature on other GBS cases related to SARS-CoV-2 infection.

## Case Report

A 57-year-old man developed dysgeusia, cough, and fever of up to 39°C lasting for 5 days. At 12 days after the resolution of the symptoms, he complained of numbness and tingling in the feet and, a few days later, also in the hands. Over 10 days, the patient developed distal limb weakness and severe gait impairment, so he was referred to the emergency department. A neurological examination showed weakness in the dorsiflexion of the foot and the extension of the toes [Medical Research Council (MRC) score: 3/5 on the right side and 4/5 on the left side], weakness in the extension of hand and fingers (MRC score: 4/5 bilaterally), gait ataxia, loss of touch and vibration sensation in the feet and ankles, weak tendon reflexes in the upper and the lower limbs, but absent ankle jerk reflex. The cranial nerves were spared. The chest radiography was negative for pneumonia, and a nasopharyngeal swab testing for SARS-CoV-2 with real-time polymerase chain reaction assay (RT-PCR) was negative, too.

**Figure 1:**
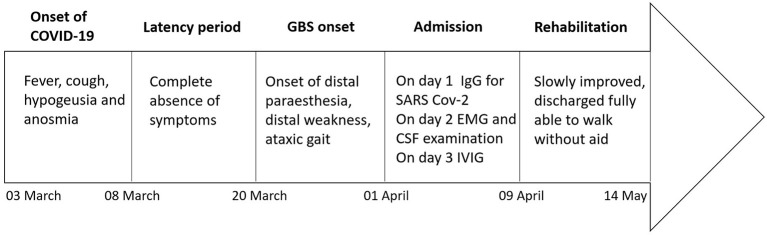
Timeline of clinical events, diagnostic-therapeutic approach, and clinical outcome.

At this stage, the patient was admitted to our unit for further diagnostic workup. The nerve conduction studies, performed 4 weeks after the neurologic onset, showed reduced or absent compound muscle action potentials and sensory nerve action potentials in the lower limbs, absent F wave response in the lower limbs, and prolonged F wave response in the upper limbs. The electromyography showed very rich spontaneous activity (fibrillation potentials and positive sharp waves) in the lower limb muscles (Table 1). The cerebrospinal fluid (CSF) examination disclosed normal cell count and normal proteins, normal CSF/serum albumin ratio, and absence of oligoclonal banding. Serum SARS-CoV-2 IgG was detected (Maglumi, Snibe). Anti-GM1, anti-GD1b, and anti-GQ1b IgG and IgM were negative (ELISA, Bühlmann). The laboratory investigations demonstrated high C-reactive protein (18.9 mg/dl). The serological tests for HIV, syphilis, cytomegalovirus (CMV), Epstein–Barr virus (EBV), and *Mycoplasma pneumoniae* (MP) were negative, except for anti-EBV, anti-CMV, and anti-MP IgG. An intravenous immunoglobulin (IVIG) cycle at 0.4 g/kg/day over 5 days was started, leading to a significant improvement of the weakness in the upper limbs and the left foot but a poor benefit on the right foot and gait ataxic. The patient was then transferred to the rehabilitation unit. He slowly improved through physiotherapy and, after 1 month, he was able to walk without aid and was discharged. Figure 1 shows a timeline of the clinical milestones of the patient.

**Table 1:** Neurophysiological findings.

**Antidromic sensory NCS**	**Latency (ms)**	**Amplitude (μV)**	**Velocity (m/s)**	**Motor NCS**	**Latency proximal/distal (ms)**	**Amplitude proximal/distal (mV)**	**Velocity (m/s)**
Sural: posterior ankle	R = NE; L = NE	R = NE; L = NE	R = NE; L = N	Tibial: medial malleolus-abductor hallucis brevis; popliteal fossa-medial malleolus	R = NE; L = NE	R = NE; L = NE	R = NE; L = NE
Radial: thumb	R = 4.2; L = NE	R = 0.13; L = NE	R = 31; L = NE				
Ulnar: digit 5	R = 2.3; L = 2.6	R = 6.2; L = 1.5	R = 52.2; L = 46.2	Common peroneal: ankle-extensor digitorum brevis; below fibula-ankle	R = 11.9/4; L = NE	R = 0.1/0.1; L = NE	R = 36.7; L = NE

*Electromyography showed fibrillation potentials and positive sharp waves in the tibialis anterior and gastrocnemius medialis muscles, bilaterally*.

*L, left; NCS, nerve conduction study; NE, not evocable; R, right*.

## Discussion

We reported a patient showing a stepwise progression of numbness, tingling, and weakness 12 days after the resolution of fever, cough, and dysgeusia. The clinical features and the electrophysiological findings along with the epidemiological context and the presence of IgG to SARS-CoV-2 supported the diagnosis of post-COVID-19 GBS. In particular, the neurophysiological examination was consistent with an acute motor-sensory axonal GBS (AMSAN) variant, with level 2 diagnostic certainty for GBS according to the Brighton Criteria (consistent clinical features and supporting nerve conduction study, but not CSF) (4, 5). Active SARS-CoV-2 infection was excluded by a complete recovery of the typical antecedent symptoms, absence of the viral genome in the nasopharyngeal swab, and negative chest radiography. The detection of serum IgG to SARS-CoV-2 is in line with the chronological profile of the antibody appearance. Indeed IgG seroconversion in COVID-19 patients is reached within a median of 13 days from the clinical onset (6).

More than half of GBS cases appear 1 to 2 weeks after an underlying infection. *Campylobacter jejuni* is the most frequent precipitant of GBS, but viral infections, including EBV,CMV, and Zika virus, are also frequently reported (7). The association of GBS with other coronaviruses was described only in two cases (8, 9). Recently, SARS-CoV-2 has also been related to GBS and Miller Fisher syndrome (MFS), wherein an autoimmune post-infectious mechanism, such as molecular mimicry or bystander activation, targeting self-ganglioside epitopes in spinal roots and peripheral nerves, might be involved, in analogy with all the other post-infectious cases.

We carried out a literature search in MEDLINE *via* PubMed for all articles published using the keywords or MeSH terms “COVID-19” or “SARS-CoV-2,” together with “Guillain–Barre syndrome,” “GBS,” “AIDP,” “AMAN,” “AMSAN,” “Miller Fisher syndrome,” or “MFS.” At the time of writing this manuscript, we found in literature 29 articles reporting 33 patients with GBS and five cases of MFS associated with SARS-CoV-2 infection, which are summarized in Table 2 (10–38).

**Table 2:** Guillain–Barré syndrome (GBS) cases related to SARS-CoV-2.

**Patient**	**Time between events**	**Clinical features**	**EMG**	**CSF**	**MRI**	**Treatment/outcome**	**References**
**GBS cases**							
77 years old, w	7 days	Tetraplegia, areflexia, paresthesia in upper limbs, facial diplegia, dysphagia, tongue weakness, and respiratory failure	AMSAN	ACD	Spine: enhancement of caudal nerve roots	Two cycles of IVIG; poor outcomes	(10)
23 years old, m	10 days	Facial diplegia, areflexia, lower limbs paresthesia, and ataxia	AMSAN	ACD	Brain: enhancement of facial nerve bilaterally	IVIG, slow improvement	(10)
55 years old, m	10 days	Tetraparesis, areflexia, paresthesia in all limbs, facial diplegia, and respiratory failure	AMAN	ACD	Spine: enhancement of caudal nerve roots	Two cycles of IVIG, poor outcomes	(10)
76 years old, m	5 days	Tetraparesis, areflexia, and ataxia	AIDP	N	Brain and spine: N	IVIG, slow improvement	(10)
61 years old, m	7 days	Tetraplegia, areflexia, lower limb paresthesia, facial diplegia, dysphagia, and respiratory failure	AIDP	ACD	Spine: N	IVG, PE, poor outcomes	(10)
61 years old, w	8 days^a^	Tetraparesis, areflexia, and sensory loss in all limbs	AIDP	ACD	n.a.	IVIG, rapid improvement	(11)
60 years old, m	20 days	Tetraparesis, areflexia, and ipopallesthesia in lower limbs, paresthesia in all limbs, facial diplegia, hypophonia, and dysarthria	AIDP	N	Cervical spine: N	IVIG, slow improvement	(12)
43 years old, m	14 days	Paraparesis, areflexia, apallesthesia, and sensory deficit in all limbs; ataxia and right peripheral facial	AIDP	ACD	Brain: multiple cranial neuritis Spine: radiculitis, and brachial and lumbar plexitis	IVIG, rapid improvement	(13)
70 years old, w	7 days	Tetraparesis, areflexia, paresthesia in all limbs, and perioral, left peripheral facial palsy, respiratory failure.	AIDP	ACD	n.a.	IVIG, rapid improvement	(13)
72 years old, m	7 days	Tetraparesis, neck flexor weakness, paresthesia, sensory loss in all limbs, respiratory failure, dysautonomia, and SIADH	AIDP	ACD	n.a.	IVIG, poor outcomes	(14)
55 years old, m	20 days	Facial diplegia, hyporeflexia, dysphagia, bilateral masseter weakness, and dysphonia	AIDP	N^b^	Brain: N	IVIG, rapid improvement	(15)
60 years old, m	20 days	Tetraparesis, areflexia and massive dysautomia (gastroplegia, paralytic ileus, and hypotension)	AMSAN	N^b^	n.a.	IVIG, rapid improvement	(15)
65 years old, m	14 days	Tetraparesis, facial diplegia, areflexia, hypopallestesia and sensory deficit in lower limbs	AMSAN	n.a.	Brain and Cervical spine: N	IVIG, n.a	(16)
70 years old, w	24 days	Tetraparesis, areflexia, distal paresthesia in all limbs and respiratory failure	AIDP	ACD	n.a.	IVIG; poor outcomes	(17)
53 years old, w	n.a^a^	Paraparesis, areflexia, paresthesia in lower limbs, dysarthria and jaw weakness	AIDP	ACD	Spine: radiculitis of cervical and lumbar spine	PE, slow improvement	(18)
76 years old, w	8 days	Tetraparesis, areflexia, distal paresthesia sensory loss in lower limbs, dysphonia, dysphagia and respiratory failure	n.a	n.a	n.a	IVIG, death	(19)
52 years old, w	15 days	Tetraparesis, areflexia, distal paresthesia and sensory loss, ataxia, dysautonomia and respiratory failure	AIDP	ACD	Spine: N	IVIG, poor outcomes	(20)
63 y years old, w	7 days	Tetraparesis, areflexia, and distal paresthesia	AIDP	N	n.a.	IVIG, slow improvement	(20)
61 y years old, w	22 days	Tetraparesis, areflexia, hypopallestesia and allodynia, facial diplegia, dysphagia, and dysautonomia	AIDP	ACD	Spine: lumbosacral nerve root enhancement	IVIG, slow improvement	(20)
50 years old, m	28 days	Paraparesis, areflexia, distal paresthesia, hypopallestesia in lower limbs, ataxic gait and facial diplegia	AIDP	N	Brain: N	IVIG, rapid improvement	(21)
54 years old, m	10 days	Tetraparesis, areflexia, paresthesia and respiratory failure	n.a.	n.a.	Spine: N	IVIG, slow improvement	(22)
58 years old, m	20 days	Facial diplegia, areflexia and paresthesia in lower limbs, dysarthria with labial sounds	AIDP/Blink reflex absent	ACD	Brain: bilateral facial enhancement	IVIG, slow improvement	(23)
68 years old, m	10 days	Tetraparesis, areflexia, sensory loss in lower limbs and respiratory failure	AIDP	ACD	Spine: N	IVIG, PE, slow improvement	(24)
57 years old, m	7 days	Tetraparesis, areflexia, paresthesia, hypopallestesia and sensory loss in lower limbs, dysphagia and respiratory failure	AIDP	ACD	n.a.	IVIG, slow improvement	(25)
64 years old, m	11 days	Tetraparesis, areflexia, paresthesia in all limbs, hypo/apallesthesia in all limbs, dysphagia and respiratory failure.	AIDP	ACD	n.a	IVIG, n.a	(26)
70 years old, w	3 days	Tetraplegia, areflexia, paresthesia in all limbs, bilateral positive Lasègue sign.	AMSAN	ACD	n.a	IVIG, n.a	(27)
43 years old, man	10days	Tetraparesis, areflexia, sensory loss in all limbs, facial diplegia and dysphagia	AIDP	n.a.	n.a.	IVIG, rapid improvement	(28)
70 years old, m	10 days	Paraparesis, areflexia, urinary retention and constipation	AIDP	ACD	Spine: N	IVIG, rapid improvement	(29)
71 years old, m	7 days	Tetraparesis, areflexia, paresthesia and hypesthesia in all limbs, respiratory failure	AIDP	ACD	n.a.	IVIG, death	(30)
64 years old, m	23 days	Paraparesis, areflexia, ipopallesthesia and sensory loss in all limbs	AIDP	ACD	n.a.	IVIG, rapid improvement	(31)
54 years old, w	21 days	Paraparesis, areflexia, paresthesia in all limbs and dysphagia	AIDP	ACD	n.a.	IVIG, rapid improvement	(32)
66 years old, w	10 days	Tetraparesis and areflexia	AIDP	ACD	n.a.	IVIG, rapid improvement	(33)
55 years old, w	15 days	Tetraparesis, areflexia, paresthesia in all limbs and perioral, facial diplegia, dysphonia, dysphagia and respiratory failure	AIDP	ACD	Brain: lepto-meningeal enhancement in medulla	IVIG, slow improvement	(34)
**Miller Fisher syndrome cases**							
50 years old, m	5 days	Ophthalmoparesis, areflexia, perioral paresthesia and ataxia	n.a	ACD	n.a.	IVIG, rapid improvement	(35)
39 years old, m	3 days	Ophthalmoparesis, areflexia	n.a	ACD	n.a.	Acetaminophen, n.a.	(35)
36 years old, m	4 days	Ophthalmoparesis (CN III and CN VI), ataxia, and hyporeflexia and sensory loss in lower limbs	n.a.	n.a.	Brain: enlargement, prominent enhancement CN III	IVIG, rapid improvement	(36)
54 years old, m	14 days	Ophthalmoparesis, tetraparesis, areflexia, distal paresthesia, facial diplegia, dysautonomia and respiratory failure	AIDP	n.a.	Spine: N	IVIG, slow improvement	(37)
74 years old, w	15 days	Ataxia and areflexia.	Increase F-wave latencies	ACD	n.a.	IVIG, rapid improvement	(38)

*ACD, albumin-cytological dissociation; AIDP, acute inflammatory demyelinating polyneuropathy; AMAN, acute motor axonal neuropathy; AMSAN, acute motor-sensory axonal neuropathy; CSF, cerebrospinal fluid; EMG, electromyography; GBS, Guillain–Barré syndrome; IVIG, intravenous immunoglobulin; m, man; MFS, Miller Fisher syndrome; MRI, magnetic resonance imaging; N, normal investigation; n.a., not available; PE, plasma exchange; w, woman*.

a *GBS manifestation precedes COVID-19*.

b *Those cases have low serum albumin, 2.9 and 2.6 mg/dl, respectively. Furthermore, a mirror-pattern oligoclonal banding was observed in both cases*.

The age of the patients ranged between 23 and 77 years (mean ± standard deviation: 59 ± 12), with a male prevalence (63.2%). The severity of COVID-19 manifestations, defined as mild, severe, and critical, according to a previously described classification (39) was as follows: mild in 30/38 (78.9%), severe in 5/38 (13.2%), and critical in 3/38 (7.9%). The time elapsed from onset of the COVID-19 symptoms to the clinical GBS manifestations ranged between 3 and 28 days (mean 12 ± 6). Notably, the timing of the majority of cases was consistent with the parainfectious profile rather than a post-infectious paradigm. In two patients, the onset of GBS actually preceded by a few days the first manifestations of COVID-19, but an earlier presentation of COVID-19 characterized by very mild or even absent symptoms could be taken into account in both cases.

The main clinical, electrophysiological, and CSF features of the patients so far reported are summarized in Table 3. With regard to GBS subtypes, the main clinical variant was the classical sensory-motor GBS (30/38), the second phenotype was MFS (5/38), the third was featured by facial diplegia with sensory deficits (2/38), and in only one case the pharyngeal–cervical–brachial variant was observed.

**Table 3:** Clinical, neurophysiological, and CSF features of Guillain–Barré syndrome/Miller Fisher syndrome cases.

**Clinical features**	***N***	**%**
Tetraparesis	24/38	63.2
Paraparesis	7/38	18.4
Ophthalmoparesis	4/38	10.5
Hypo/areflexia	38/38	100
Facial weakness	15/38	39.5
Paresthesia and/or sensory loss	30/38	78.9
Ataxia	9/38	21.1
Bulbar	11/38	28.9
Dysautonomia	6/38	15.8
Respiratory failure	15/38	39.5
SIADH	1/38	2.6
**NCS**		
AIDP	26/38	68.4
AMSAN	5/38	13.2
AMAN	1/38	2.6
n.a.	5/38	13.2
Only F wave delay	1/38	2.6
**CSF**		
ACD	25/38	65.8
Mirror OB	2/38	5.3
Normal	5/38	13.2
n.a.	6/38	15.8

*ACD, albumin-cytological dissociation; AIDP, acute inflammatory demyelinating polyneuropathy; AMAN, acute motor axonal neuropathy; AMSAN, acute motor-sensory axonal neuropathy; NCS, nerve conduction studies, CSF, cerebrospinal fluid; OB, oligoclonal band; n.a., not available; SIADH, syndrome of inappropriate antidiuretic hormone secretion*.

Following the first reported cases of GBS related to SARS-CoV-2 (10), a more common axonal rather than demyelinating variant has been suggested. However, unlike the initial reports, the electrophysiological features in other cases did not show a higher prevalence of axonal variants in these patients. By contrast, the demyelinating and the mixed forms were more often observed.

The examination of CSF samples obtained from 32 patients showed an albumin-cytological dissociation in 68.4% of cases. All RT-PCRs for SARS-CoV-2 on CSF were negative, suggesting the lack of a direct causative role of the virus.

Anti-ganglioside antibodies were detected only in two cases of MFS, showing a borderline positivity for IgM anti-GM1 and a positivity for IgG anti-GD1b, respectively.

Brain and/or spinal cord MRI was performed in 20 patients and showed contrast enhancement of nerve roots at the level of the cauda equina, of brachial and lumbosacral plexus, and also of single or multiple cranial nerves. In addition, a brainstem and cervical leptomeningeal enhancement, which is an atypical feature in GBS, was seen in one case.

Almost all patients were treated with IVIG and/or plasma exchange, whereas one mild case received only symptomatic treatment. The recovery timing and the outcomes varied widely, but in 14/38 cases, a rapid improvement was reported. In 12/38 cases, the improvement was instead slower and required admission to rehabilitation facilities, whereas 6/38 patients had a poor outcome (prolonged stay in the intensive care unit and long-lasting severe disabilities). Two cases were fatal, and in 4/38 cases, the outcome was not available. Of relevance is the fact that it does not seem that COVID-19 severity at onset is correlated with GBS outcomes.

Moreover, the clinical features of post-COVID-19 GBS did not differ from those of cases related to other viruses, with the notable exception of a remarkable respiratory involvement (Table 3). It is indeed crucial to highlight that respiratory failure was present in 15 /38 cases (39.5%) of GBS related to SARS-CoV-2, a percentage higher than that observed in previous GBS cohorts (ranging from 20 to 30%) (5). This suggests that COVID-19 pneumonia may overlap with GBS-associated respiratory muscle weakness and increase the number of cases needing a respiratory support. The reported observations indicate that physicians should always consider GBS in the differential diagnosis of a respiratory insufficiency in COVID-19 patients, especially in cases of normocapnic or hypercapnic respiratory failure (pointing to a restrictive respiratory pattern in contrast with the interstitial pattern of COVID-19 pneumonia) or when a discrepancy between chest imaging and respiratory parameters occurs. An additional explanation for this latter scenario is that the respiratory failure in GBS associated with COVID-19 may also be driven by a dysfunction of the cardiorespiratory centers in medulla oblongata directly induced by the virus (40) since the SARS-CoV-2 genome has also been detected in the human brainstem (2).

The early recognition of GBS symptoms is critical, given the associated high mortality as well as severe motor disabilities that may seriously limit the quality of life of these patients (41). Deficits induced by this neurological condition could be reasonably included among the post-COVID-19 sequelae, which requires an accurate evaluation by the neurologists, similarly to what has been suggested in the past outbreaks, for instance, in cases of post-polio syndrome (42).

## Conclusion

Our case report and review of literature contribute to raise awareness of the possible association between GBS and SARS-CoV-2 infection. The underlying mechanism of injury could be an autoimmune reaction against peripheral nerve antigens, in light of the lack of a viral genome in the CSF.

The main clinical, electrophysiological, and CFS features of the patients so far reported proved to be similar to GBS cases related to other infectious diseases. Nevertheless, respiratory involvement is more frequent in GBS related to SARS-CoV2, and a reasonable explanation for this finding could be the coexistence of COVID-19 interstitial pneumonia and GBS respiratory muscle weakness since the majority of cases had a parainfectious profile.

However, the relationship between SARS-CoV-2 and GBS, actually described only in single case reports and small case series, should be confirmed in larger observational studies in order to evaluate the temporal correlation between GBS clusters and the COVID-19 epidemic curve in each affected country.

## Data Availability Statement

The raw data supporting the conclusions of this article will be made available by the authors, without undue reservation.

## Ethics Statement

Written informed consent was obtained from the patient for the publication of this case report, including any potentially identifiable images or data included in this article.

## Author Contributions

AZ, MC, and AC were involved in the work-up of the patient, planning and conducting investigations, and providing clinical care. AZ planned the case report and drafted the initial manuscript. EA and MT performed the electrophysiological investigation. DF and MG carried out the laboratory testing. AZ, MT, and MCo reviewed the literature. EA, DF, MG, MT, MCo, MC, and AC revised the manuscript. All the authors approved the final manuscript as submitted.

## Dedication

This paper is dedicated to the loving memory of our colleague, Prof. Arrigo Moglia, neurologist and neurophysiologist at our Institute, who recently died from COVID-19.

## Conflict of Interest

The authors declare that the research was conducted in the absence of any commercial or financial relationships that could be construed as a potential conflict of interest.
